# Behavioral and Sociodemographic Factors Associated with Vision-Related Quality of Life in Keratoconus: A Cross-Sectional Study in Riyadh, Saudi Arabia

**DOI:** 10.3390/vision10030041

**Published:** 2026-07-05

**Authors:** Seham Alsalamah, Yara Abosabaah, Ghadah Alhabs, Lujain Marghlani, Mahmood Showail, Taghreed Alnahedh, Mohammed Taha

**Affiliations:** 1 College of Medicine, King Saud bin Abdulaziz University for Health Sciences, Riyadh 3660, Saudi Arabia; yara.hamed071@gmail.com (Y.A.);; 2 King Abdullah International Medical Research Center (KAIMRC), Riyadh 3660, Saudi Arabia; 3 Department of Ophthalmology, Faculty of Medicine, King Abdulaziz University, Jeddah 80200, Saudi Arabia; 4 Department of Ophthalmology, King Abdulaziz University Hospital, King Abdulaziz University, Jeddah 80200, Saudi Arabia; 5 Academic Affairs, College of Medicine, King Saud bin Abdulaziz University for Health Sciences, Riyadh 3660, Saudi Arabia; 6 Optometry Department, King Abdullah International Medical Research Center (KAIMRC), National Guard Health Affairs, Riyadh 3660, Saudi Arabia; 7 Department of Ophthalmology, College of Medicine, King Saud bin Abdulaziz University for Health Sciences, Riyadh 3660, Saudi Arabia

**Keywords:** keratoconus, vision-related quality of life, NEI-VFQ-25, Saudi Arabia, corneal disease, quality of life

## Abstract

Keratoconus is a progressive corneal ectasia that results in substantial visual impairment and imposes a significant functional and psychosocial burden on affected individuals. Despite evidence suggesting earlier onset and a potentially more aggressive disease course of keratoconus in Saudi populations, there is a significant lack of region-specific data evaluating vision-related quality of life (VRQoL) in Riyadh. This cross-sectional study conducted in Riyadh, Saudi Arabia, evaluated VRQoL in individuals with keratoconus using the 25-item National Eye Institute Visual Function Questionnaire (NEI-VFQ-25). Data were obtained from January to March 2025 through an online, structured, self-administered questionnaire. A total of 1007 participants were included, of whom 299 (29.7%) reported a diagnosis of keratoconus. The mean composite NEI-VFQ-25 score among participants with keratoconus was 61.0 ± 15.8, reflecting moderate impairment in VRQoL. Reduced VRQoL was significantly associated with eye-rubbing behavior, including both frequency (*p* = 0.027) and method (*p* = 0.026). The results highlight the importance of early detection and the relevance of eye-rubbing behavior in relation to reduced VRQoL, while supporting the need for longitudinal research to further clarify these associations.

## 1. Introduction

Keratoconus is a progressive, non-inflammatory corneal disorder characterized by cone-like protrusion of the cornea, irregular stromal thinning, and significant visual loss. While the etiology of keratoconus remains unclear, it is known to be associated with certain systemic conditions such as Down syndrome, Leber congenital amaurosis, and mitral valve prolapse [1]. Keratoconus typically manifests in the second to third decade of life and usually affects both eyes. It is clinically classified as mild, moderate, or severe, with progressive stages presenting with symptoms such as blurred or distorted vision and increased corneal steepening [2].

Several studies have investigated vision-related quality of life (VRQoL) in patients diagnosed with keratoconus. A study conducted in Saudi Arabia involving 429 patients found that individuals aged 30 years or younger, females, and those not using visual aids scored lower on the National Eye Institute Visual Function Questionnaire-25 (NEI-VFQ-25), indicating poorer quality of life [3]. Similarly, a Belgian study analyzing 77 keratoconic eye pairs reported that both high and low optical aberrations were significantly associated with VRQoL, surpassing even high-contrast visual acuity in predictive strength [4].

As with many ocular disorders, keratoconus significantly impacts VRQoL [5]. Its assessment relies on patient-reported outcomes and encompasses multiple domains, including visual functioning, symptoms, emotional well-being, social relationships, concerns, and convenience [6]. This makes VRQoL a comprehensive and patient-centered indicator. Although general VRQoL instruments may not fully capture all disease-specific visual disturbances related to irregular astigmatism and higher-order optical aberrations, prior evidence has shown that both higher- and lower-order optical aberrations are significantly associated with VRQoL in keratoconus and may predict VRQoL more strongly than high-contrast visual acuity [4]. Nevertheless, the NEI-VFQ-25 remains a validated and widely used tool for assessing broad patient-reported visual functioning across ophthalmic conditions, allowing comparison with previous keratoconus VRQoL studies [7].

Eye rubbing is a clinically relevant behavioral factor in keratoconus. A large systematic review and meta-analysis of keratoconus prevalence and risk factors identified eye rubbing among the most important risk factors for the disease. In a study of patients with keratoconus or ocular surface disease, higher eye-rubbing behavioral scores were associated with more frequent and intense ocular symptoms. These findings support the relevance of eye rubbing as a risk factor for keratoconus and a behavior linked to symptom burden and patient-reported outcomes [8,9].

Assessing keratoconus-related VRQoL in Saudi Arabia is particularly important, as the disease in this population tends to present earlier and progress more severely than in other ethnic groups [10,11]. This variation highlights the importance of regional research, as findings from other populations may not be applicable. Regional variation may also be relevant because low humidity has been reported to have a significant and biologically plausible association with increased keratoconus prevalence, with prevalence increasing by approximately 3% for every 1% decrease in relative humidity [12]. In Saudi Arabia, inland regions, including Riyadh, experience arid conditions with relative humidity below 25%, while the driest central regions reach relative humidity levels below 10–15% [13]. Preliminary studies, including one by Al-Dairi et al., reported a mean NEI-VFQ-25 score of 58.6 among Saudi patients, with color vision scoring highest (82.5) and mental health scoring lowest (44.7) [3]. However, no study to date has specifically evaluated VRQoL among keratoconus patients in Riyadh or examined, within a Saudi population, the association between eye-rubbing behavior and VRQoL. Therefore, this cross-sectional study aims to evaluate keratoconus-related VRQoL in Riyadh, Saudi Arabia and examine its association with sociodemographic factors and eye-rubbing behavior.

## 2. Materials and Methods

This cross-sectional study was conducted in the Riyadh region of Saudi Arabia. Data were obtained from January to March 2025 using an online, structured, self-administered questionnaire created using Google Forms (Google LLC, Mountain View, CA, USA) and distributed to the general population. Duplicate responses were prevented by enabling the Google Forms setting that limits responses to one submission per respondent. A non-probability convenience sampling approach was used, whereby participants were recruited based on accessibility through online distribution of the questionnaire, including targeted outreach to relevant communities and support groups. The survey link was disseminated through social media platforms, and participation was voluntary. As the questionnaire was distributed through open online channels, a formal response rate could not be calculated. The required sample size was estimated using the Epitools online calculator (https://epitools.ausvet.com.au/; accessed on 14 February 2024) assuming a 50% proportion, 5% precision, and a 95% confidence level. Eligible participants were adults aged ≥18 years of either sex who completed the questionnaire. Participants were included irrespective of keratoconus status, and subgroup analyses were subsequently performed among those with a self-reported diagnosis of keratoconus by an ophthalmologist. No exclusion criteria were applied, and all individuals meeting the inclusion criteria were enrolled.

**Figure 1:**
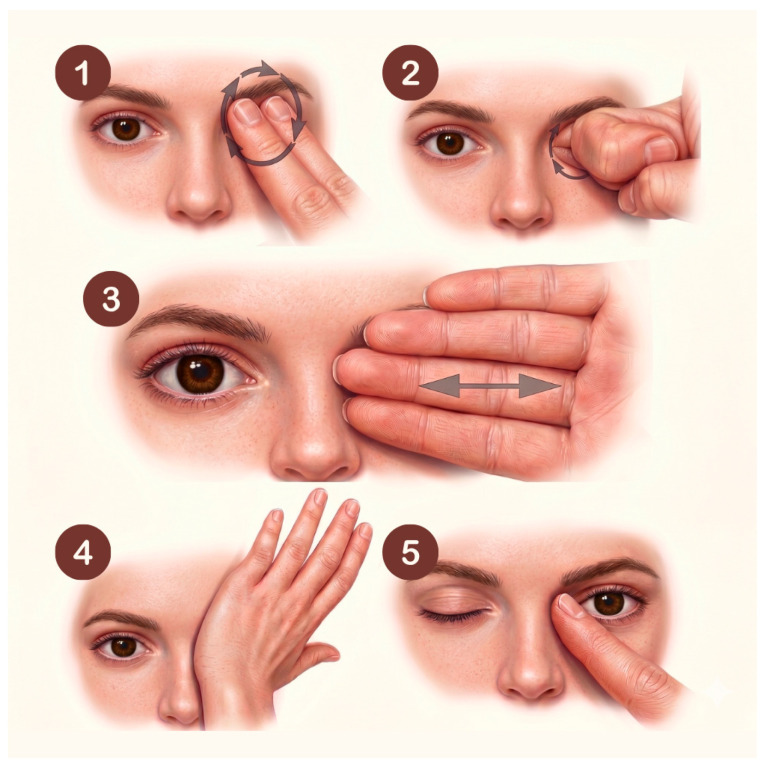
Visual representations of common eye-rubbing techniques used in the questionnaire to assess eye-rubbing behavior.

The first component of the questionnaire captured sociodemographic variables including age, gender, marital status, educational attainment, employment status, and income level. Behavioral factors related to eye rubbing, including frequency and technique, were also assessed. Eye-rubbing technique was evaluated using textual descriptions of commonly reported rubbing patterns, complemented by an illustrative visual aid to enhance participant understanding (Figure 1). The visual aid was generated using Gemini (Google), a generative artificial intelligence model, and was informed by eye-rubbing techniques previously described in the literature [14,15]. Figure 1 includes five distinct techniques: (1) two-finger rotation/fingertips; (2) knuckle; (3) back of the hand horizontal movement; (4) palm; and (5) finger pad on the corner of the eye.

The second component consisted of the National Eye Institute Visual Function Questionnaire-25 (NEI-VFQ-25), administered in its validated Arabic version [10]. The NEI-VFQ-25 is a widely utilized instrument that evaluates patient-reported vision-related quality of life across 12 domains: general health, general vision, ocular pain, near activities, distance activities, social functioning, mental health, role difficulties, dependency, driving, color vision, and peripheral vision. Items are scored on a 0–100 scale, where higher scores reflect better visual function. Difficulty-based items are scored from 100 (“no difficulty”) to 0 (“stopped due to eyesight”), while frequency-based items range from 100 (“none of the time”) to 0 (“all of the time”). Responses marked as “not applicable” were excluded from scoring. The questionnaire was distributed and completed online to ensure accessibility and consistency in data collection. The primary outcome was the composite NEI-VFQ-25 score, which reflects the overall vision-related quality of life. Secondary outcomes included individual subscale scores and their relationships with sociodemographic, behavioral, and clinical variables [16].

Descriptive statistics were used to summarize participant characteristics. Subscale scores were calculated as the mean of all valid responses within each domain. The overall composite score was calculated as the average of all vision-related subscales, excluding the general health domain, while the driving subscale was included only for participants who reported driving. Associations between categorical variables were examined using the Chi-square test or Fisher’s exact test, as appropriate. Comparisons between continuous variables were conducted using the independent sample *t*-test and one-way analysis of variance (ANOVA). All analyses were performed using IBM SPSS Statistics for Windows, Version 29.0.0 (IBM Corp., Armonk, NY, USA). A two-tailed *p* value of <0.05 was considered statistically significant.

## 3. Results

### 3.1. Sociodemographic Characteristics

Of the 1007 participants initially enrolled, 299 reported a physician diagnosis of keratoconus and were included in the VRQoL analysis. Table 1 summarizes the sociodemographic characteristics of the full study cohort (*N* = 1007). The sample included participants from several sociodemographic categories, with a predominance of young adults and university-educated individuals. Additional demographic and clinical characteristics are summarized in Table 1.

### 3.2. Eye Rubbing and Keratoconus Profile

Eye rubbing behavior was common among participants, with most individuals (46.8%; *n* = 471) reporting at least occasional rubbing. The most frequently reported method involved using the finger pad at the corner of the eye, followed by fingertip or rotational techniques. Among the 299 participants with self-reported physician-diagnosed keratoconus, 38.79% (*n* = 116) had lived with the diagnosis for more than 10 years, while 47.16% (*n* = 141) reported a family history of keratoconus. Detailed distributions of eye rubbing behaviors and keratoconus-related characteristics are presented in Table 2.

### 3.3. Vision-Related Quality of Life Scores

Across the keratoconus group, the mean NEI-VFQ-25 composite score was 61.0 ± 15.8, indicating moderate impairment in vision-related quality of life. The most severely affected subdomains included ocular pain, role difficulties, and dependency, reflecting a substantial psychosocial and functional burden, with mental health also notably impacted. In contrast, higher scores were observed in color vision, peripheral vision, and general health, suggesting that certain visual functions remain relatively preserved (Table 3).

### 3.4. Quality of Life by Participant Characteristics

When comparing NEI-VFQ-25 scores across demographic and clinical variables, age was significantly associated with VRQoL (*p* = 0.022), with the highest mean score in the 21–40 years group (62.11 ± 15.35) and the lowest in those aged >60 years (44.24 ± 19.06). Significant associations were identified between VRQoL and both the frequency and method of eye rubbing. Participants who never rubbed their eyes reported the highest VRQoL scores (65.03 ± 14.83), while those who always rubbed their eyes reported the lowest (57.17 ± 16.58). Individuals using knuckle or palm techniques for rubbing had lower scores compared to other methods. No statistically significant differences were observed in relation to gender, educational level, employment status, chronic illness, allergic history, or consanguinity (Table 4).

## 4. Discussion

Keratoconus is a corneal disorder characterized by cone-like protrusion and stromal thinning, leading to irregular astigmatism and visual impairment in the affected population [1]. The etiology remains unclear, although multiple studies have reported associations with genetic syndromes [17]. The condition typically manifests in young adults and often affects both eyes with varying degrees of severity [2]. Assessing VRQoL in these patients is essential, as it encompasses not only functional vision but also emotional and social well-being. Given regional variations in disease burden and presentation, our study aimed to evaluate VRQoL in keratoconus patients in Riyadh, Saudi Arabia, using the NEI-VFQ-25 instrument.

Keratoconus prevalence varies widely across populations, with global estimates ranging up to 5% depending on geographic region and diagnostic method [18]. A recent systematic review by Mohaghegh et al. (2023) estimated the global prevalence of keratoconus to be approximately 1.40 per 1000 population [19]. In Riyadh, Almudhaiyan et al. (2020) reported a keratoconus prevalence of 9.89% among 400 Saudi adults aged 20–40 years using Scheimpflug tomography and Amsler–Krumeich criteria [20]. Building on this regional evidence, the present study was designed to evaluate keratoconus-related VRQoL rather than estimate disease prevalence; therefore, the proportion of keratoconus respondents reflects the study focus and should not be interpreted as a population-based prevalence estimate. Notably, 45.1% of participants reported parental consanguinity, consistent with prior evidence suggesting an increased occurrence of keratoconus in consanguineous populations. Afzal et al. (2023) demonstrated a higher prevalence of keratoconus among offspring of consanguineous parents compared to those of unrelated parents [21]. In our cohort, nearly half of participants (47.16%) reported a family history of keratoconus, aligning with prior evidence that underscores the hereditary contribution to disease susceptibility [22].

Eye rubbing behaviors were significantly associated with VRQoL. Participants who never rubbed their eyes had the highest QoL scores, whereas those who reported “always” rubbing had the lowest. This is consistent with findings by Najmi et al. (2019), who identified habitual eye rubbing as a risk factor for both disease progression and ocular discomfort [23]. The method of eye rubbing was also associated with VRQoL as patients who used aggressive techniques such as knuckles or palms had worse scores. Previous studies similarly emphasized the role of mechanical trauma in corneal ectasia [24,25]; however, in the present study, eye rubbing may also represent a marker of ocular discomfort, allergy, worse visual quality, or greater disease burden, particularly because visual acuity, disease severity, and corneal tomography were not assessed.

The mean VRQoL score among keratoconus patients was 61.0 ± 15.8, suggesting moderate impairment. Dairi et al. (2024) reported a comparable score of 58.6 ± 18.0 [3]. Subscales with the lowest scores included ocular pain (38.3), role difficulties (41.1), and dependency (47.4). These findings align with earlier literature indicating that keratoconus affects activities requiring fine visual acuity, such as reading and driving [26]. Zabadi et al. (2023) similarly demonstrated that keratoconus impairs near and distance vision, general vision, mental health, and social functioning, although no significant differences were observed in ocular pain, role difficulties, or dependency [27].

Mental health scores were low (mean: 48.4), indicating reduced mental health-related VRQoL, as captured by the NEI-VFQ-25 mental health subscale. This finding is consistent with prior literature suggesting that keratoconus may be associated with emotional and psychological distress [28]. Significant impairments were also observed across multiple subscales, particularly in near activities, peripheral vision, and distance activities. Additionally, prior evidence suggests that irregular astigmatism and corneal thinning may distort visual perception, including color [29]. Age was also significantly associated with VRQoL, with the lowest mean NEI-VFQ-25 scores observed among participants older than 60 years. Although this finding was statistically significant, it should be interpreted cautiously because the >60 years subgroup included only 14 participants. Lower VRQoL in older participants may partly reflect unmeasured age-related ocular comorbidities, visual acuity differences, disease severity, or treatment history rather than keratoconus alone.

This study has several limitations. First, its cross-sectional design restricts the ability to draw causal inferences between keratoconus and VRQoL; therefore, the observed associations should be interpreted cautiously. Second, the analysis was limited to univariate comparisons, and subgroup analyses were not adjusted for multiple testing, which may increase the risk of residual confounding and Type I error. Third, keratoconus status was based on self-reported physician diagnosis without independent clinical or imaging confirmation, which may have introduced reporting or misclassification bias. In addition, the online convenience-based sampling strategy may have introduced selection bias, particularly if individuals with greater visual symptoms or psychosocial burden were more likely to participate. Finally, clinical variables such as visual acuity, disease severity or staging, corneal tomography, corrective modality, contact lens wear, and prior interventions were not captured and may represent unmeasured confounders influencing VRQoL.

Despite these limitations, this study provides considerable insights into VRQoL in patients with keratoconus and, to the best of our knowledge, represents one of the largest questionnaire-based studies evaluating VRQoL in this population in Saudi Arabia. The findings highlight potential areas of importance for early screening, particularly among individuals at higher risk of keratoconus, and support the relevance of eye-rubbing behavior in relation to reduced VRQoL. Given the observed impact on functional and psychosocial domains, routine assessment of VRQoL may help identify individuals who could benefit from additional support.

Future research should employ longitudinal and multicenter designs to clarify the direction of the observed associations and improve generalizability. Objective diagnostic confirmation, disease severity measures, visual function data, corrective modalities, and treatment history should be incorporated to better characterize the clinical determinants of VRQoL. Multivariable analyses with appropriate adjustment for multiple comparisons would further help identify independent predictors and strengthen the reliability of subgroup findings.

## 5. Conclusions

Keratoconus is associated with reduced VRQoL, particularly affecting visual function, mental health, and role performance. Lower VRQoL scores were observed in relation to both the frequency and method of eye rubbing. These findings highlight key associations and may inform future research and awareness efforts, while underscoring the need for longitudinal studies to further clarify these relationships.

## Figures and Tables

**Table 1:** Sociodemographic Characteristics of Participants (*N* = 1007).

		Frequency *N* (%)
Gender	Female	488 (48.5%)
	Male	519 (51.5%)
Age (Years)	<20 Years	327 (32.5%)
	21–40 Years	527 (52.3%)
	41–60 Years	139 (13.8%)
	>60 Years	14 (1.4%)
	Mean (SD)	28.4 (10.9)
Marital Status	Single	658 (65.3%)
	Married	312 (31.0%)
	Widow/Divorced	37 (3.7%)
Educational Level	High School Level or Less	267 (26.5%)
	University Level or More	740 (73.5%)
Employment Status	Unemployed/Student	635 (63.1%)
	Employed	372 (36.9%)
Family Income	<5K SAR	231 (22.9%)
	5–10K SAR	267 (26.5%)
	10–20K SAR	303 (30.1%)
	>20K SAR	206 (20.5%)
Chronic Disease	No	787 (78.1%)
	Yes	220 (21.9%)
Allergic Disorders	No	757 (75.2%)
	Yes	250 (24.8%)
Family History of Allergy	No	536 (53.2%)
	Yes	471 (46.8%)
Parental Consanguinity	No	553 (54.9%)
	Yes (1st Cousin)	268 (26.6%)
	Yes (2nd Cousin)	186 (18.5%)

Abbreviations: SD: Standard deviation; SAR: Saudi Arabian riyal.

**Table 2:** Eye-Rubbing Behaviors and Keratoconus Diagnosis Among Participants (*N* = 1007).

		Frequency *N* (%)
Eye Rubbing Frequency	Never	57 (5.7%)
	Rarely	215 (21.4%)
	Sometimes	471 (46.8%)
	Often	185 (18.4%)
	Always	79 (7.8%)
Eye Rubbing Method	Finger Pad on Corner of Eye	278 (27.6%)
	Two-Finger Rotation/Fingertips	240 (23.8%)
	Knuckle	216 (21.4%)
	Palm	187 (18.6%)
	Back of the Hand Horizontal Movement	61 (6.1%)
	All Above	25 (2.5%)
Keratoconus Diagnosis by an Ophthalmologist	No	708 (70.3%)
	Yes	299 (29.7%)
Duration Since Keratoconus Diagnosis (*n* = 299)	<1 Years	36 (12.04%)
	2–4 Years	87 (29.10%)
	5–9 Years	60 (20.07%)
	>10 Years	116 (38.79%)
Family History of Keratoconus (*n* = 299)	Yes	141 (47.16%)

**Table 3:** Descriptive Statistics of NEI-VFQ-25 Subscales Among Participants with Keratoconus (*n* = 299).

VFQ Subscale	Mean (SD)
General Health	72.6 (25.0)
General Vision	65.2 (26.5)
Ocular Pain	38.3 (15.1)
Near Activities	68.5 (24.2)
Distance Activities	65.2 (26.1)
Vision Specific	
Social Functioning	64.4 (26.9)
Mental Health	48.4 (22.4)
Role Difficulties	41.1 (23.8)
Dependency	47.4 (31.6)
Driving	69.7 (28.7)
Color Vision	77.3 (26.8)
Peripheral Vision	74.1 (28.5)
Overall Score	61.0 (15.8)

Abbreviations: NEI-VFQ-25: National Eye Institute Visual Functioning Questionnaire-25; VFQ: Visual Functioning Questionnaire; SD: Standard deviation.

**Table 4:** Stratified Analysis of Vision-Related Quality of Life by Sociodemographic and Clinical Factors in Patients with Keratoconus (*n* = 299).

		VRQoL Mean (SD)	Sig. Value
Gender	Female	60.24 (15.47)	0.452 ^a^
	Male	61.62 (16.14)	
Age	<20 Years	61.02 (9.33)	0.022 ^b^
	21–40 Years	62.11 (15.35)	
	41–60 Years	57.01 (19.25)	
	>60 Years	44.24 (19.06)	
Marital status	Single	61.64 (14.27)	0.666 ^b^
	Married	60.62 (17.67)	
	Widow/Divorced	58.02 (16.67)	
Educational level	High School Level or Less	57.43 (14.65)	0.052 ^a^
	University Level or More	61.86 (16.00)	
Employment status	Unemployed/Student	60.56 (15.49)	0.633 ^a^
	Employed	61.44 (16.23)	
Family Income	<5K SAR	61.50 (15.26)	0.587 ^b^
	5–10K SAR	59.90 (15.73)	
	10–20K SAR	59.94 (17.07)	
	>20K SAR	63.61 (15.24)	
Chronic Diseases	No	61.93 (15.17)	0.115 ^a^
	Yes	58.63 (17.04)	
Allergic Disorders	No	61.85 (15.80)	0.151 ^a^
	Yes	59.00 (15.75)	
Family Allergic History	No	61.13 (15.43)	0.866 ^a^
	Yes	60.82 (16.23)	
Parental Relation	No	60.52 (15.85)	0.575 ^b^
	Yes (1st Cousin)	62.36 (16.52)	
	Yes (2nd Cousin)	59.87 (14.60)	
Eye Rubbing Frequency	Never	65.03 (14.83)	0.027 ^b^
	Rarely	62.24 (16.80)	
	Sometimes	61.09 (15.39)	
	Often	58.11 (15.64)	
	Always	57.17 (16.58)	
Eye Rubbing Method	Finger Pad on Corner of Eye	63.52 (16.73)	0.026 ^b^
	Two-Finger Rotation/Fingertips	61.33 (14.70)	
	Knuckle	56.45 (16.93)	
	Palm	56.20 (12.99)	
	Back of the Hand Horizontal Movement	62.16 (13.09)	
	All Above	66.22 (12.74)	

Abbreviations: NEI-VFQ-25: National Eye Institute Visual Functioning Questionnaire-25; QoL: Quality of life; SD: Standard deviation; SAR: Saudi Arabian riyal. Statistical tests: ^a^ Independent samples *t*-test; ^b^ One-way ANOVA.

## Data Availability

The data presented in this study are available on request from the corresponding author. The data are not publicly available due to privacy and ethical restrictions.

## Abbreviations

The following abbreviations are used in this manuscript:

IRB	Institutional Review Board
KAIMRC	King Abdullah International Medical Research Center
MNGHA	Ministry of National Guard Health Affairs
NEI-VFQ-25	National Eye Institute Visual Function Questionnaire-25
QoL	Quality of life
SAR	Saudi Arabian riyal
SD	Standard deviation
VFQ	Visual Function Questionnaire
VRQoL	Vision-related quality of life
